# An integrative pan cancer analysis of RET aberrations and their potential clinical implications

**DOI:** 10.1038/s41598-022-17791-y

**Published:** 2022-08-17

**Authors:** Lei Zhou, Juanni Li, Xiaofang Zhang, Zhijie Xu, Yuanliang Yan, Kuan Hu

**Affiliations:** 1 Department of Anesthesiology, Third Xiangya Hospital of Central South University, Changsha, 410008 Hunan China; 2 Department of Pathology, Xiangya Hospital, Central South University, Changsha, 410008 Hunan China; 3 National Clinical Research Center for Geriatric Disorders, Xiangya Hospital, Central South University, Changsha, 410008 Hunan China; 4 Departments of Burn and Plastic, Ningxiang People’s Hospital, Hunan University of Chinese Medicine, Changsha, 410600 Hunan China; 5 Department of Pharmacy, Xiangya Hospital, Central South University, Changsha, 410008 Hunan China; 6 Department of Hepatobiliary Surgery, Xiangya Hospital, Central South University, Changsha, 410008 Hunan China

**Keywords:** Cancer, Genetics

## Abstract

RET (rearranged during transfection), encoding a tyrosine kinase receptor, is a novel therapeutic target for cancers. The aberrations of RET are commonly found in cancers. Here, we profiled a comprehensive genomic landscape of RET mutations, copy number variants (CNVs), co-occurrence of RET and its mRNA expression and methylation levels in pan cancer, paving the way to the development of new RET-targeted therapies in clinic. Analysis of RET somatic mutations, CNVs, co-occurrence, mRNA expression and methylation were performed among 32 cancer types from The Cancer Genome Atlas (TCGA) dataset covering a total of 10,953 patients with 10,967 samples. RET aberrations were found in 3.0% of diverse cancers. The top two RET-altered tumors were skin cutaneous melanoma (SKCM) and uterine corpus endometrial carcinoma (UCEC) with dominant mutations in the other and PKinase_Tyr domains. RET-G823E and RET-S891L were most commonly found in SKCM and UCEC. Thyroid carcinoma (THCA) demonstrated the highest rate of coiled-coil domain containing 6 (CCDC6)-RET fusions, which constitutively activate RET kinase. Two FDA-approved RET inhibitors—pralsetinib and selpercatinib have been implied for the treatment of patients with RET S891L mutant UCEC and the treatment of patients with metastatic RET-fusion positive THCA and non-small cell lung cancer (NSCLC) at therapeutic level 1. We also identified four RET M918T-altered cases in patients with pheochromocytoma and paraganglioma (PCPG), which may induce drug resistance against multikinase inhibitors. Next, 273 co-occurring aberrations, most frequently in Notch signaling, TGF-β pathway, cell cycle, and Ras-Raf-MEK-Erk/JNK signaling, were uncovered among 311 RET altered cases. TP53 mutations (162 patients) leads to the most significant co-occurrence associated with RET aberrations. Furthermore, the RET expression was found most significantly increased in breast invasive carcinoma (BRCA) and neck squamous cell carcinoma (HNSC), as compared to their corresponding normal tissues. At last, patients with higher expression and sequence variant frequency have a worse prognosis, such as sarcoma patients. This work provided a profound and comprehensive analysis of RET and co-occurred alterations, RET mRNA expression and the clinical significance in pan cancer, offering new insights into targeted therapy for patients with RET anomalies.

## Introduction

Rearrangement during transfection (RET) was first identified as a chimeric proto-oncogene that underwent rearrangement in a classic NIH-3T3 cells transformation assay^1^. The human *RET* gene, located in chromosome 10 (10q11.2), encodes a transmembrane receptor tyrosine kinase composed of an intracellular domain, transmembrane domain, cysteine-rich region and an N-terminal extracellular domain^2^. RET, highly expressed in neurons, dopamine, peripheral enteric and ureteric bud, is required for the development of kidneys and neurons. Binding to growth factors, the RET receptor changes its phosphorylation status and therefore results in the activation of downstream signaling, including PI3K and MAPK pathways. Gain- and loss-of-function of RET are associated with many diseases, such as hirschsprung disease (HD), medullary thyroid carcinoma and multiple endocrine neoplasias type 2^3–5^. A recent study has reported that there were 88 RET-altered cases among 4,871 cancer patients (approximately 2%) based on clinical grade high throughput next-generation sequencing, however, most are unclearly oncogenic^6^. Of note, the missense mutation, fusion protein, amplifications and rearrangements are the most common RET alterations.

Although the majority of RET alterations are of unknown significance, RET has been considered as a novel therapeutic target in various cancers. Several inhibitors with anti-RET activity have been approved by FDA, such as sunitinib for the treatment of renal cell carcinoma and neoplasms, sorafenib, lenvatinib and vandetanib for the treatment of differentiated thyroid cancer subtypes^5,7^. Meanwhile, therapies against RET aberrations have shown promising clinical responses^8–11^. There are so far two RET-selective inhibitors (selpercatinib and pralsetinib) that have been granted accelerated approval by the FDA. Selpercatinib, in particular, is FDA-approved for the treatment of patients with metastatic RET-altered medullary thyroid cancer and adult patients with metastatic RET fusion-positive NSCLC^12,13^.

In this study, we profiled a comprehensive landscape of RET mutation, CNVs, mRNA expression and methylation levels among 32 different cancer types derived from The Cancer Genome Atlas (TCGA) database. To facilitate the development of clinical trials targeting RET aberrations, we also analyzed the correlation between RET alterations and patient prognosis in diverse cancers. Collectively, this study highlights the crucial role of RET in tumorigenesis and provides new insights in targeting RET alterations for cancer therapy.

## Results

### RET somatic mutations across diverse cancers

**Figure 1:**
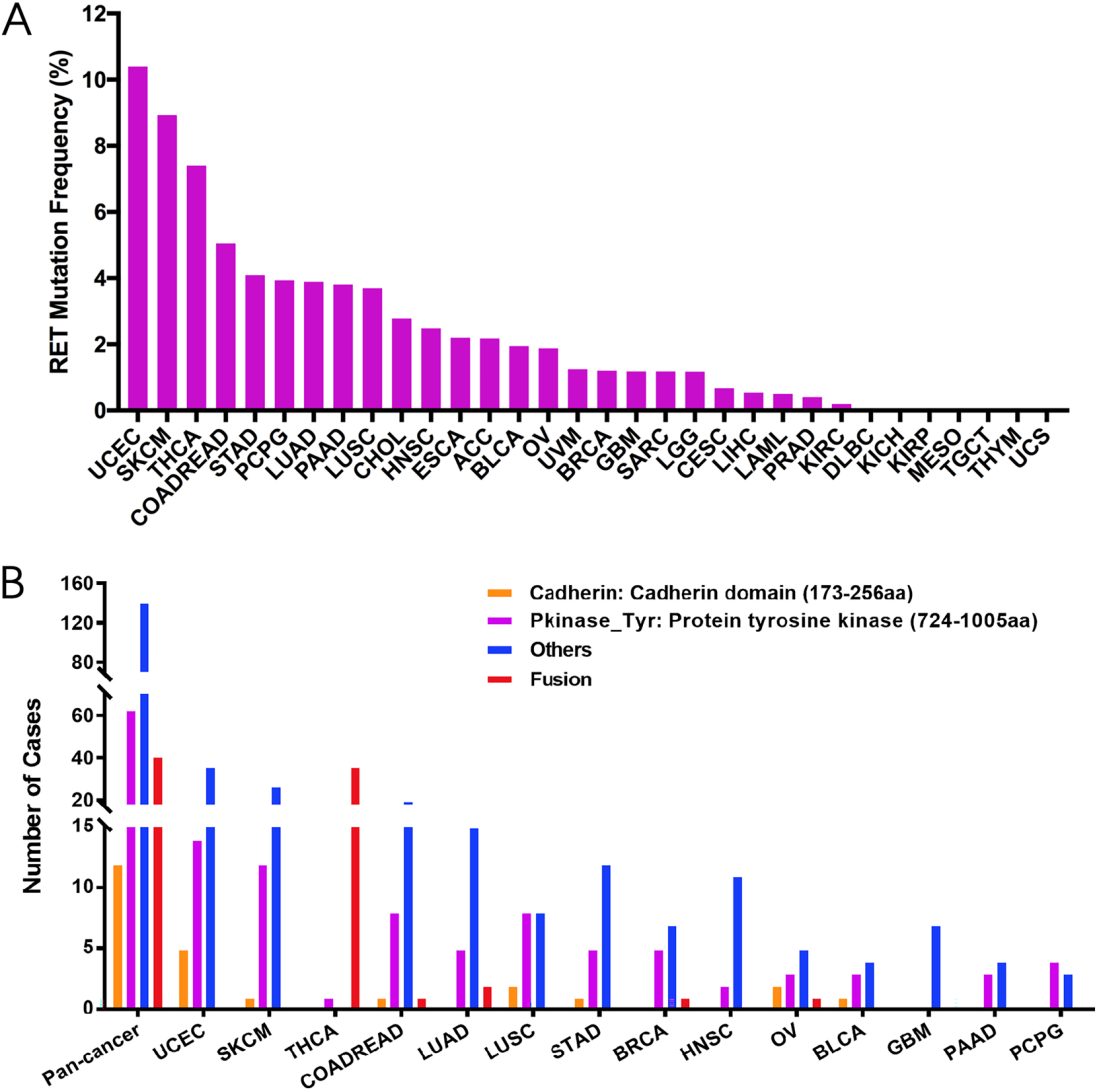
The frequency and distribution of RET somatic mutation across diverse TCGA cancers. (**A**) The frequency of RET somatic mutation in 32 cancer types. (**B**) The distribution of RET somatic mutation within different functional domains in pan cancer and the top 10 cancers. RET functional domains: cadherin domain (173-256 aa), protein tyrosine kinase domain (PKinase_Tyr, 724-1005 aa) and other domains. *Aa* amino acid.

Somatic alterations in the *RET* gene have been indicated in multiple nonhereditary cancers. A total of 311 cases with mutant RET was identified among 10,967 samples with the given 32 cancer types (Supplementary Tables S1, S2). Specifically, RET variants frequencies were the highest in uterine corpus endometrial carcinoma (UCEC, 10.4%), skin cutaneous melanoma (SKCM, 8.93%), thyroid carcinoma (THCA, 7.40%), colon and rectal adenocarcinomas (COADREAD, 5.05%), stomach adenocarcinoma (STAD, 4.09%), lung adenocarcinoma (LUAD, 3.89%), and lung squamous cell carcinoma (LUSC, 3.70%) (Fig. 1A). In contrast, some types of cancers including, but not limited to, kidney renal papillary cell carcinoma (KIRP), tenosynovial giant cell tumor (TGCT), thymoma (THYM) and mesothelioma (MESO) were barely detected with any RET mutations. Those containing too few cases might not represent the accurate status of RET aberrations (Fig. 1A, Supplementary Table S2).

RET contains three functional domains: an extracellular cadherin-like domain (173–256 aa), a hydrophobic transmembrane domain, and an intracellular protein tyrosine kinase domain (PKinase_Tyr, 724–1005 aa). After binding to specific ligands, the cadherin domain results in RET receptor dimerization and further phosphorylates tyrosine residues within the intracellular PKinase_Tyr domain^2^. In pan cancer, we identified 311 RET oncogenic alterations, including 97 mutations in two indicated functional domains, 172 mutations in other domains and 42 fusions. The distribution of all these changes varied across different cancer types. While THCA contributed to most RET fusion cases (36 cases), other cancers tended to have higher prevalence of mutations in two indicated functional domains and the others. In addition, the dominating mutations between two regions were consistently observed in the PKinase_Tyr segment across all kinds of cancers, especially UCEC (14 cases) and SKCM (12 cases) (63 out of 75, Fig. 1B, Supplementary Table S3).

Using cBioPortal cancer genomics database, the 311 RET somatic mutations were divided into four different categories based on their functional effects on protein coding. Missense mutation leads to the top one type of mutations (232 cases), followed by fusion (42 cases), truncating mutation (35 cases), and in-frame mutation (2 cases) (Supplementary Fig. S1A). The most common RET somatic mutation is M918T (exon 16) in the Pkinase_Tyr domain, which has been detected in 4 pheochromocytoma and paraganglioma (PCPG) samples (Supplementary Fig. S1B). All four M918T mutations in indicated PCPG cases are considered to be oncogenic and have level-3B therapeutic implications. Although both selective RET inhibitors -pralsetinib and selpercatinib-demonstrate superior inhibition against M918T mutant THCA, their clinical therapeutic effects on M918T mutant PCPG patients still need more investigation. It has also been suggested that the M918T mutation may lead to drug resistance, especially against the VEGFR-inhibitor motesanib, and is associated with advanced medullary thyroid cancer (MTC)^14^. Notably, there are another three identified missense mutations in PCPG—C634R, C631Y and C515S (Supplementary Fig. S1B). The RET C634R and C631Y mutations are known to be oncogenic^15,16^, however, the biological significance of RET C515S mutation is unclear. To date, the RET-targeted inhibitor selpercatinib (LOXO-292) is FDA-approved for the treatment of patients ≥ 12 years of age with metastatic RET-mutant medullary thyroid cancer who require systemic therapy and adult patients with metastatic RET fusion-positive NSCLC^17^, its clinical effect in patients with above three mutant PCPG remains unknown. UCEC demonstrated the highest frequency of RET-related alterations. But the function of most mutations was largely unknown. The top three mutations in UCEC have been determined as S891L, E511D and E768G, located in the Pkinase_Tyr domain and the other domain as described (Supplementary Fig. S1C). The RET S891L mutation has not been clinically validated and its oncogenicity remain exclusive.

**Figure 2:**
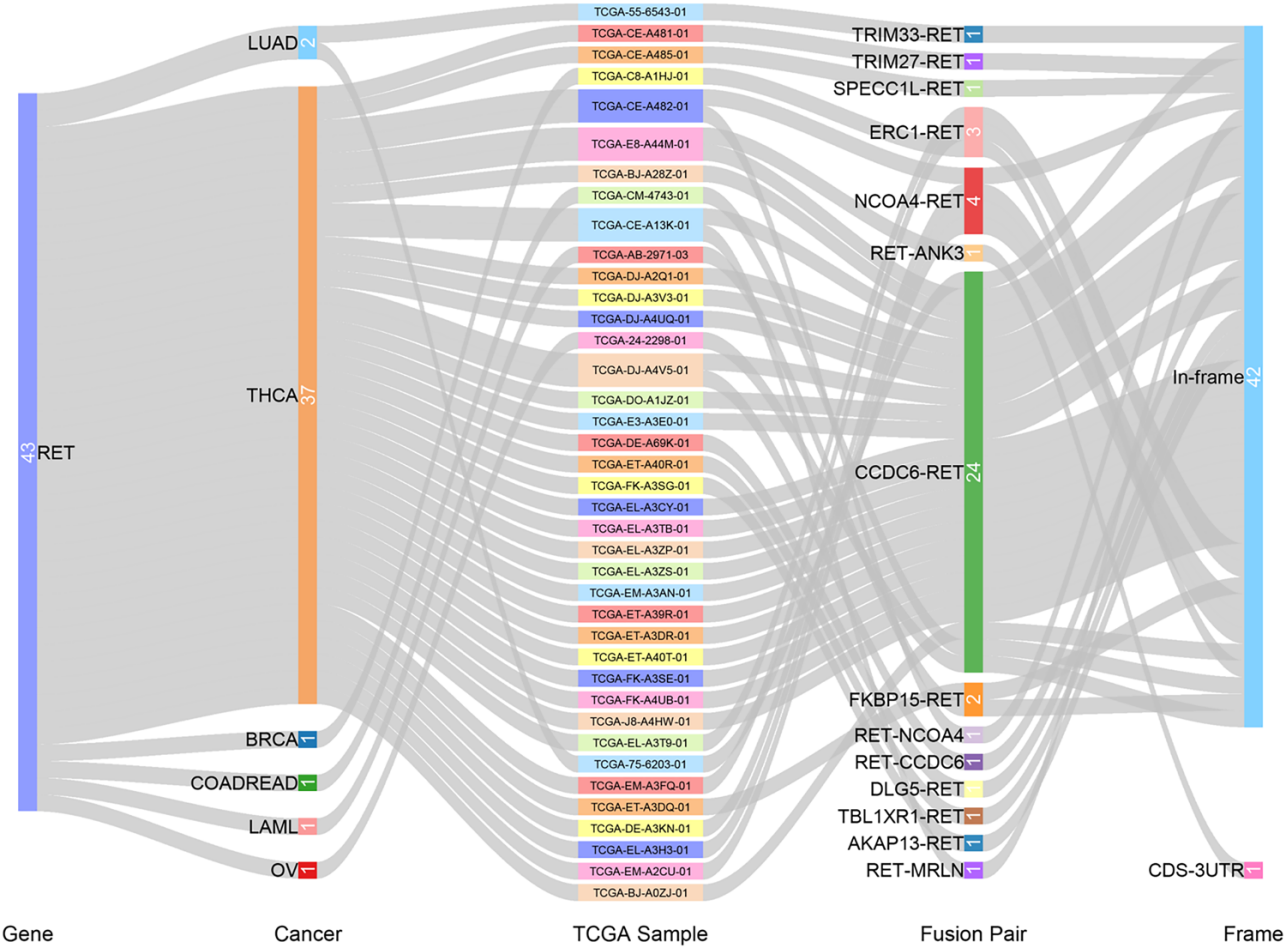
Fusion gene of RET in indicated TCGA cancer types.

Fusion genes could express high levels of active abnormal proteins and play important roles in tumorigenesis, thus they are therefore potential targets for cancer diagnostic treatment^18^. Based on both cBiopotal and TCGA Fusion Gene Databases, we were able to comprehensively determine RET fusion transcripts across multiple cancers. Interestingly, THCA demonstrated the highest frequency of RET fusion genes, including twenty-two CCDC6, five NCOA4, two ERC1, one TRIM27, one SPECC1L, one ANK3, one DLG5, one TBL1XR1, one AKAP13, two ERC1, one FKBP15 and one MRLN (Fig. 2). CCDC6, encoded by Coiled Coil Domain Containing 6 gene, was initially detected after chromosomal translocation of RET in some thyroid tumors. It constitutively activates the tyrosine kinase of RET receptor and might represent a potential predictive biomarker of tumor radio- and chemo-resistance^19^. Except the ANK3 RET fusion transcript, the rest all belong to in-frame class. There were also a few fusion genes identified in other cancer types. For example, we detected TRIM33 and CCDC6 in LUAD. TRIM33, also known as transcriptional intermediary factor 1 gama, regulates TGF-β1 receptor and has been considered as a suppressor gene in multiple cancers^20^.

**Figure 3:**
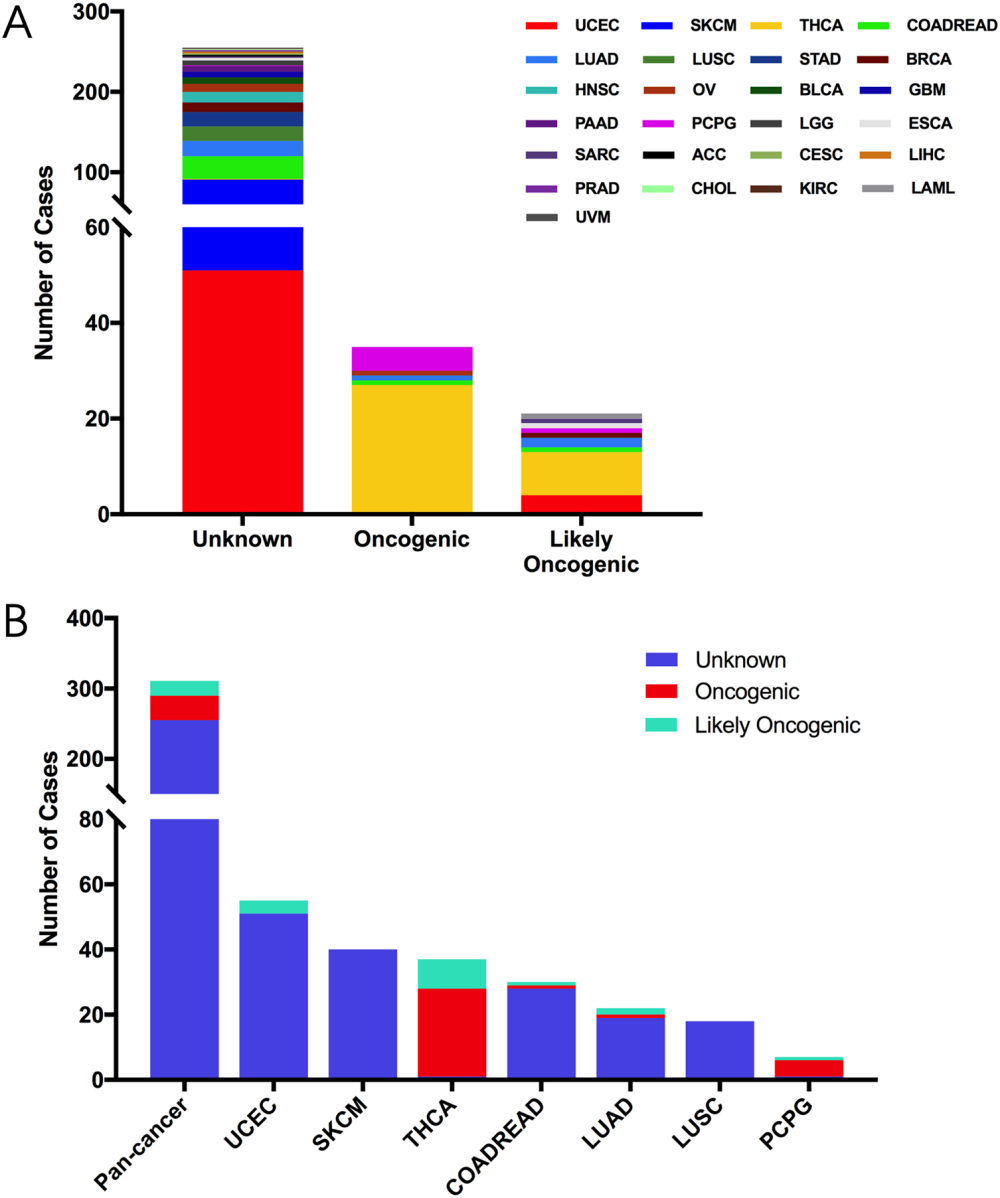
The classification of RET somatic mutation according to its functional impact. (**A**) The recapitulative classification of RET somatic mutation according to its functional impact in diverse TCGA cancers. (**B**) The functional impact level distribution of RET somatic mutation in pan cancer and the top 7 cancer types.

Based on the oncogenic effects and predictive significance, we divided total 311 somatic mutations into three categories: oncogenic (35 mutations), likely oncogenic (21 mutations) and unknown (255 mutations) (Fig. 3A). Across all cancer types, the oncogenic effects of most RET mutations remain unknown, indicating more efforts are needed in investigating the oncogenic roles of these mutations. Notably, in THCA, 27 RET fusion mutations have been identified as oncogenic mutations, while nine RET fusion with THCA are likely oncogenic (Fig. 3B). One THCA case with missense mutation in the tyrosine kinase domain has been demonstrated to be unknown mutation. THCA contributed to the most part in oncogenic category of RET mutations. Besides, in PCPG, 5 cases with missense mutations (4 in the tyrosine kinase domain and 1 in other domain) have been considered to be oncogenic aberrations.

**Figure 4:**
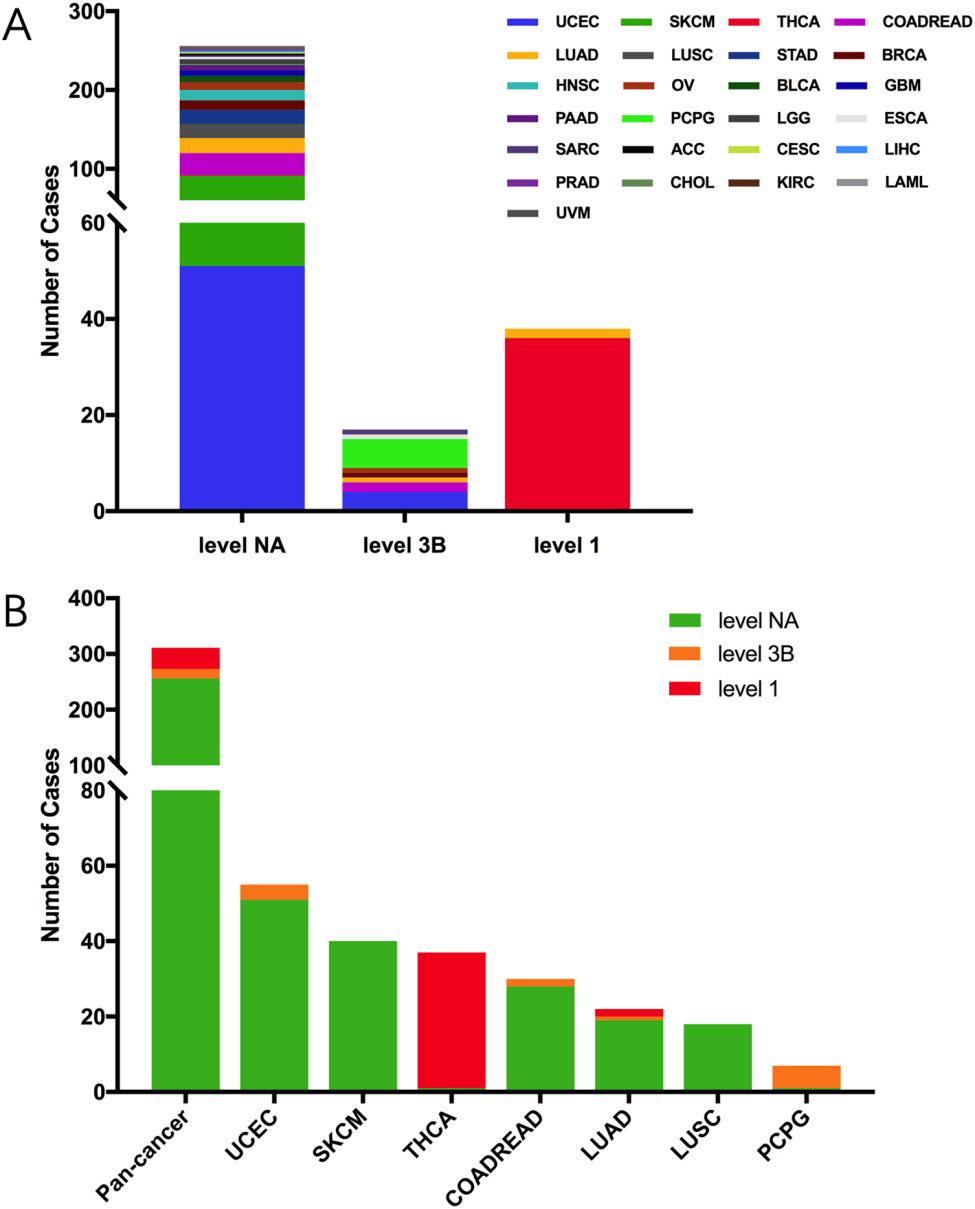
The classification of RET somatic mutation according to its therapeutic implications. (**A**) RET somatic mutation classification based on its therapeutic implications as annotated in OncoKB among diverse TCGA cancers. (**B**) Targeted therapy implications distribution of RET somatic mutations in pan cancer and the top 7 cancer types.

We further analyzed the therapeutic implications of RET mutations as defined by OncoKB classification from cBioPortal database in pan cancers. There were 3 different levels for RET mutations: level NA, level 3B and level 1^21^. Each of them contained 256 cases, 17 cases and 38 cases, respectively (Fig. 4A). Only Level 1 includes RET alterations recognized by the FDA for targeted therapy with an FDA-approved drug, such as selpercatinib and pralsetinib, while level 3B implies an investigational biomarker predictive of response to an FDA-approved or investigational drug for other indications. The level NA represents variants with unknown oncogenic effect and unclear biological effect. Interestingly, all sequence variants with Level 1 were observed only in THCA and LUAD, whereas those with level 3B were found in UCEC, COARDREAD and PCPG. In particularly, all mutations in THCA stayed at level 1, which indicated a crucial role of targeting RET mutations in clinical THCA treatment (Fig. 4B). Based on the data from cBioportal, the therapeutic levels vary not only among different RET variants but also changed across diverse cancer types. For instance, the therapeutic implication of CCDC6-RET fusion was at level 1 in THCA and LUAD, but it became level 3B in COADREAD and OV (Supplementary Table S2).

### RET copy number variation (CNV) in different cancers

**Figure 5:**
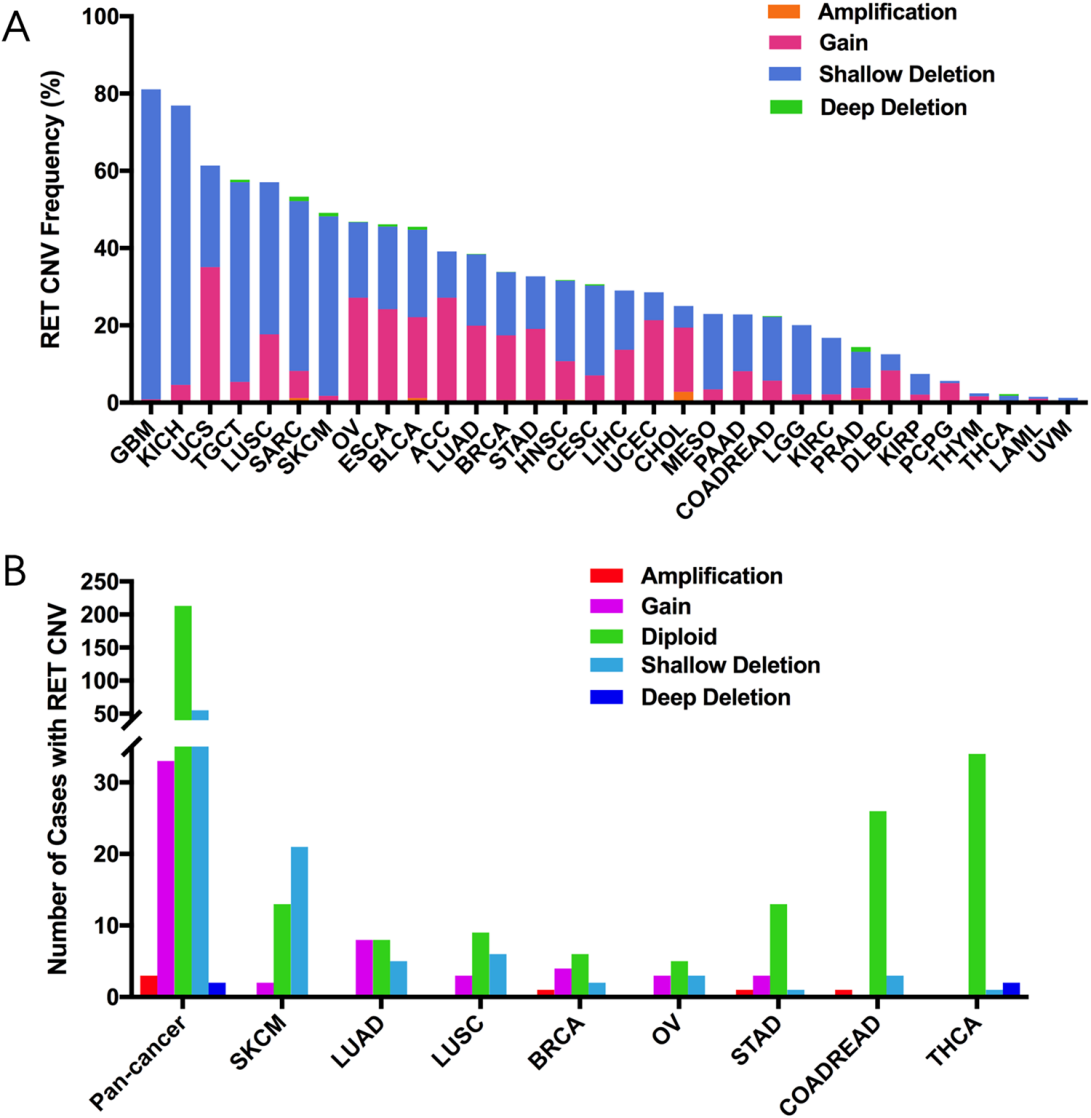
RET CNVs distribution among TCGA cancers. (**A**) The frequency of RET CNVs across 32 types of TCGA cancers. (**B**) The distribution of RET CNV in pan cancer and the top 8 cancer types. *CNVs* copy number variants.

Copy number variation (CNV) has been indicated as one of the most common forms of genetic alterations that could affect the RET expression in cancers. Here, we first performed comprehensive analysis of RET mRNA levels in 32 TCGA cancers. As shown in Supplementary Fig. S2A, RET expression not only varied among different cancers, but also exhibited a broad spectrum within certain cancers. For instance, based on the interquartile range, some cancers, such as liver hepatocellular carcinoma (LIHC), sarcoma (SARC) and COADREAD, had a widespread range of RET expression, while others—like TGCT, THCA and glioblastoma multiforme (GMB)—did not. It might indicate that some cancers had more than one subtype and therefore showed higher genetic diversity of RET. Next, we were able to identify 3630 RET CNVs in total 10,967 cancer samples using ciBioportal. Interestingly, most CNV cases were shallow deletion (2382 samples) and gain (1184 samples). In addition, the top five RET CNV frequencies were found in GBM (81.1%), kidney chromophobe (KICH) (76.9%), uterine carcinosarcoma (UCS) (61.4%), TGCT (57.7%), and LUSC (57.1%), respectively (Fig. 5A). We also investigated the relationship between the RET CNV and its mRNA level. Indeed, RET CNV positively correlated with RET expression among 32 TCGA cancers (r = 0.0931, p < 0.0001) (Supplementary Fig. S2B). This data suggested a crucial role of CNV in regulating RET expression in cancers.

We further identified 93 RET CNVs including 55 cases with shallow deletion, 33 cases with gain, 3 cases with amplification, and 2 cases with deep deletion among the above 311 cases including missense_sequence variant, fusion, frame_shift_deletion, frame_shift_insert, in_frame_deletion, nonsense_sequence variant, splice_region, and splice_site (Fig. 5B, Supplementary Table S2). SKCM and LUSC contributed to the most RET CNVs with shallow deletion (21 cases in SKCM and 6 cases in LUSC), while LUAD showed the most RET CNVs with gain (8 cases). Interestingly, SKCM had much lower RET mRNA level compared to other cancers (Supplementary Fig. S2A), indicating that shallow deletion might play an important role in inhibiting RET transcription. In contrast, acute myeloid leukemia (LAML), for example, which lacked the gain and amplification of RET, demonstrated the lowest RET mRNA level. However, although LUAD had high frequency of RET CNV with gain, its RET mRNA level remained relatively low, indicating there might be additional alterations involved in regulating the transcription of RET in cancer, such as methylation.

### RET alterations (mutation and CNVs) across diverse cancers

**Figure 6:**
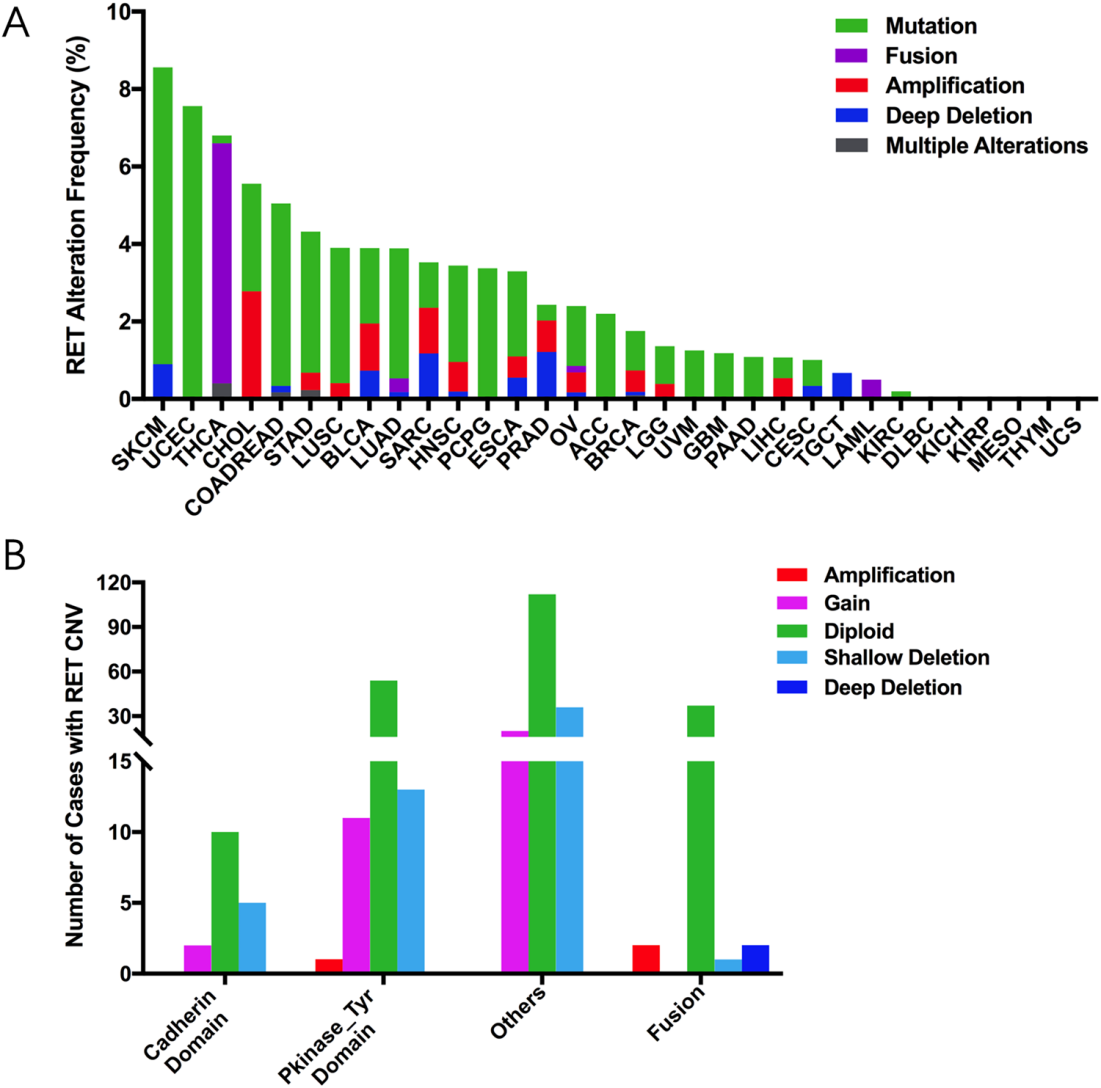
The frequency and distribution of RET alterations in TCGA cancers. (**A**) The frequency of combined RET alterations including mutations and CNVs among 32 TCGA cancers. (**B**) The distribution of RET CNVs in combination with RET mutations in diverse RET functional domains.

The combined RET mutation and CNV frequencies varied among cancers and the average frequency in pan cancer was 3.0% (333 of 10,967 samples). SKCM (8.56%), UCEC (7.56%), THCA (6.8%), cholangiocarcinoma (CHOL) (5.56%) and COADREAD (5.05%) demonstrated the highest RET alterations frequencies (Fig. 6A). The leading RET alteration form was mutation in most cancers, except THCA, CHOL, SARC and prostate adenocarcinoma (PRAD). For instance, there were 6.2% of RET fusion in THCA and 2.8% of RET amplification in CHOL. Other cancer types, such as STAD, LUSC, LUAD, head and neck squamous cell carcinoma (HNSC) and PCPG, also exhibited dominant RET mutations although they had much lower rate of RET alterations. Notably, some cancers including Lymphoid neoplasm diffuse large B-cell lymphoma (DLBC), KICH, KIRP, MESO, THYM and UCS barely had any RET alterations (Fig. 6A).

Furthermore, the distribution of CNVs varied depended on different RET mutation forms. 11 cases with RET copy gain and 13 cases with RET shallow deletion contributed to the majority of RET mutations in the Pkinase_Tyr domain. Similarly, almost all RET mutations in other domains were accompanied by gain (20 cases) and shallow deletion (36 cases). However, mutations of RET fusion had very few concurrent CNVs (Fig. 6B).

### Co-occurring aberrations associated with RET alterations in oncogenic pathways

**Figure 7:**
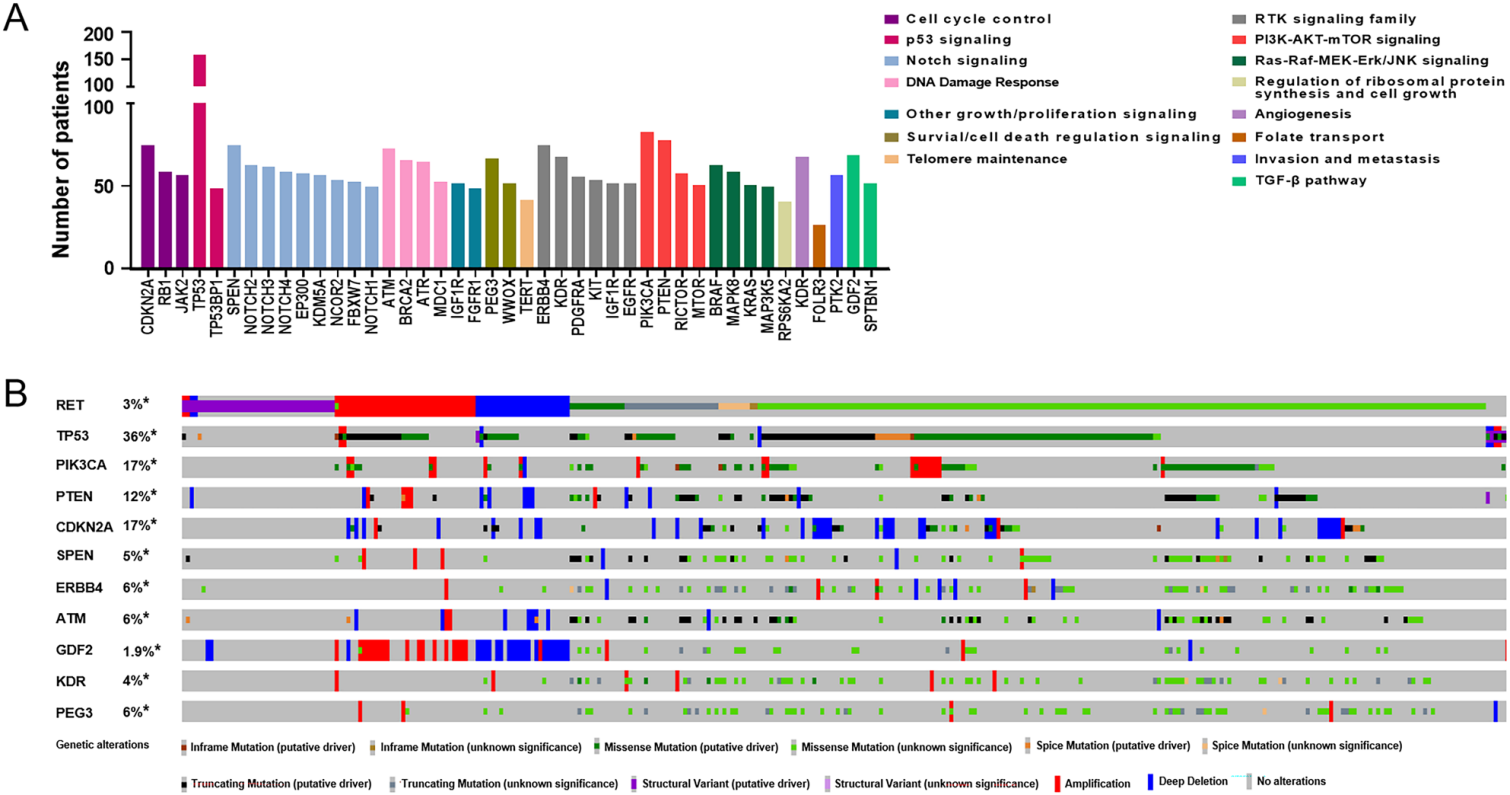
Co-occurring aberrations associated with RET alterations in oncogenic pathways in pan cancer. (**A**) Histogram visualizing the number of patients with alterations of RET and co-occurring targets in the selected oncogenic pathways. The most significant co-altered gene or co-aberrant targets found in > 50 cases in each pathway were included in the histogram. Please also see the complete list of co-occurring alterations associated with RET aberrations in individual pathway in Supplementary Table S4. (**B**) OncoPrint of genetic alterations of RET and top-ten co-occurring targets in pan cancer. Numbers represent the frequencies of indicated alterations in pan cancer. Different colors and symbols were used to distinguish different genetic alterations, such as amplifications, mutations, and deep deletions.

RET-driven cancers commonly harbor co-occurring alterations in oncogenic pathways that affect tumor biology and response to targeted therapies^22^**.** To better understand the biological roles of RET alterations in the setting of cancers, we next profiled the co-occurring mutations associated with RET alterations in diverse oncogenic pathways in pan cancer. A total of 273 significant co-aberrations associated with RET alterations were identified among the selected gene network in oncogenic pathways including—but not limited to—cell cycle, p53 signaling, Notch signaling, RTK signaling, and PI3K-AKT-mTOR signaling (Fig. 7A, Supplementary Table S3). The most common co-occurring alterations were genes involved Notch signaling (1496 cases; 47 significant genes among 55 genes), followed by TGF-β pathway (1108 cases; 42 significant genes among 43 genes), cell cycle (890 cases; 30 significant genes among 34 genes), and Ras-Raf-MEK-Erk/JNK signaling (774 cases; 24 significant genes among 26 genes), respectively. Notably, 162 RET-mutant cases concurrently harbored TP53 mutations, leading the most frequently co-occurring alterations associated with RET aberrations. In addition, the distribution of aberrations of RET and top-ten co-occurring genes, such as TP53 (n = 162; *P* < 0.001), PIK3CA (n = 84; *P* < 0.001), PTEN (n = 79; *P* < 0.001), and CDKN2A (n = 76; *P* < 0.001), were further analyzed across 32 types of TCGA cancers using OncoPrint (Fig. 7B). Interestingly, more than a half of missence RET-mutant cases with unknown significance had other co-aberrations with putative driver roles in tumorigenesis, such as TP53 truncating mutation and PIK3CA missence mutation.

### RET expression and methylation analysis in pan cancer

**Figure 8:**
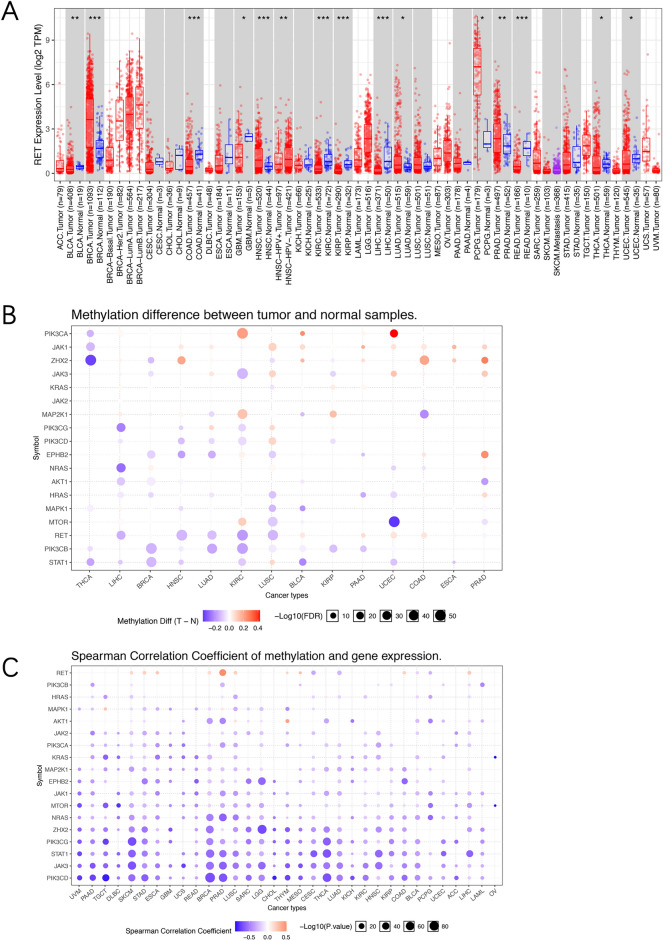
RET mRNA levels and methylation in TCGA cancers. (**A**) RET mRNA levels (log2 TPM transformed) between tumor tissues and the paired normal tissues among diverse TCGA cancers from TIMER2. (**B**) Bubble map illustrating the differences of RET and related downstream genes methylation between tumor tissues and corresponding normal tissues among cancers. Red dot: the upregulation of methylation in indicated cancers, blue dot: the downregulation of methylation in indicated cancers. (**C**) Bubble map depicting the relation between methylation and RET and its downstream genes expression in different TCGA cancers. Red dot: the increases in both methylation level and gene expression level, blue dot: the increase in methylation level but decrease in gene expression level. *TPM* transcripts per million. *p < 0.05, **p < 0.01, ***p < 0.001.

Abnormal RET expression has been indicated in various cancers^23^. Actually, the RET levels were even dramatically different among normal tissues based on the GTEx portal database. As shown in Supplementary Fig. S3, substantia nigra in brain showed the highest RET expression, while cultured fibroblasts and EBV-transformed lymphocytes had very low levels. We next compared the RET mRNA expression between tumors and matched normal tissues through TCGA database (Fig. 8A). Seven cancer types, including bladder urothelial carcinoma (BLCA), breast invasive carcinoma (BRCA), HNSC, LUAD, PCPG, PRAD and THCA, showed significantly increased RET expression compared to their corresponding normal tissues. By contrast, decreased RET levels were observed in COADREAD, GMB, LIHC, kidney renal clear cell carcinoma (KIRC), KIRP and UCEC. Notably, the RET level is much higher in HPV- HNSC tumors compare to HPV + samples. Besides, PCPG demonstrated the most upregulated RET expression (TPM) as compared to its normal tissue (TPM) (Fig. 8A).

DNA methylation is also known to regulate gene expression in cancers. Hence, we compared the RET methylation levels between TCGA cancers and paired normal tissues using GSCALite. Interestingly, the RET methylation levels were found to be downregulated in some cancers, such as LIHC, HNSC, LUAD, KIRC and LUSC (Fig. 8B). We also analyzed the correlation between RET methylation and its downstream genes expression in 32 TCGA cancers. RET methylation and mRNA levels were both increased in cancers including PRAD, SKCM, ESCA, BRCA, LUSC, THYM, MESO, COAD and LIHC. However, in cancers such as LUAD and CESC, it increased at the methylation level but decreased at the gene expression level. Interestingly, the mRNA levels of its downstream pathway mediators, such as MAPK1, JAK2 and KRAS, were found to be negatively associated with their methylation levels in most cancer types (Fig. 8C).

### RET alterations and patient survival

**Figure 9:**
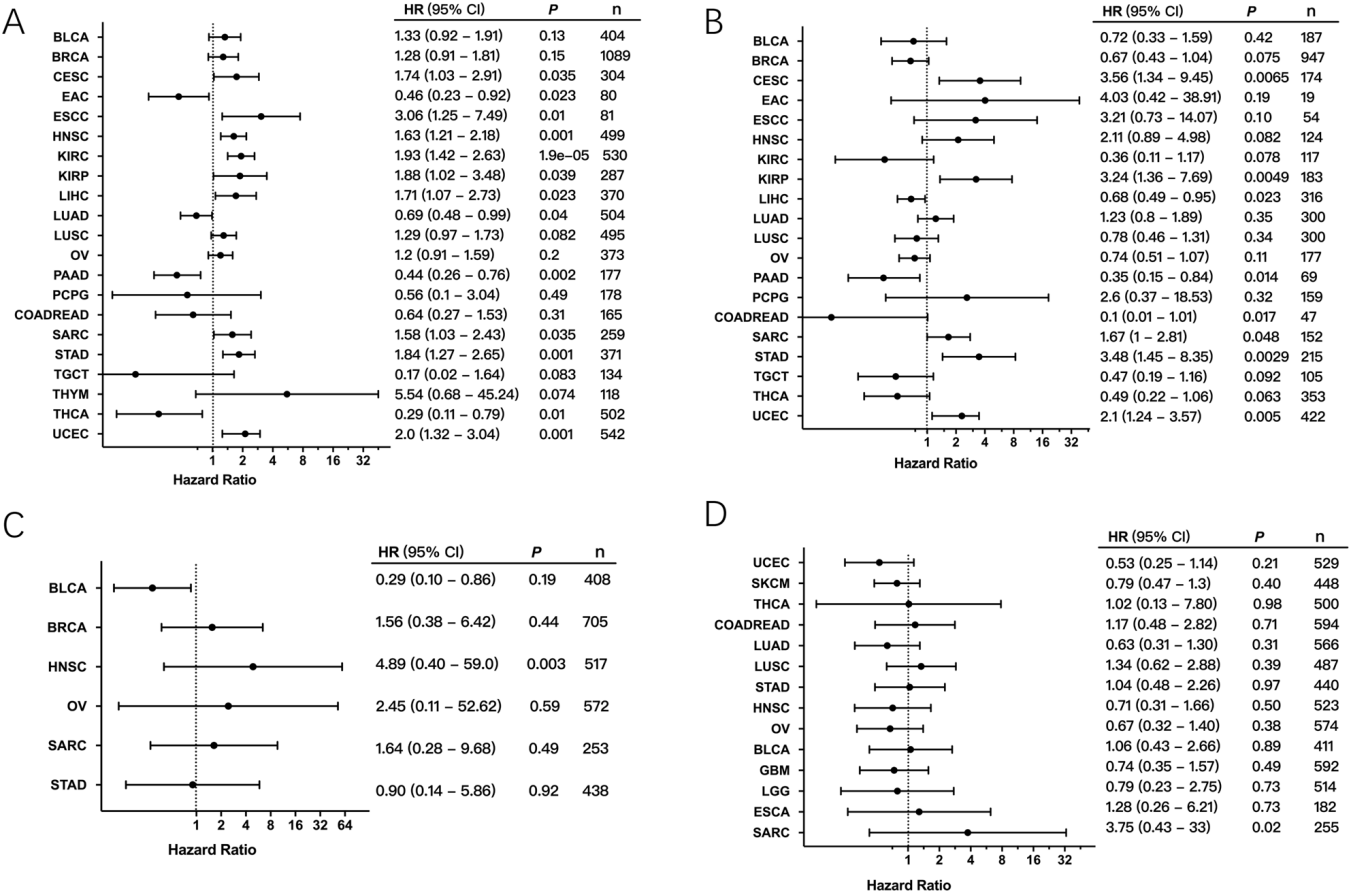
The correlation between RET alterations and patient survival. (**A**) The correlation between RET mRNA expression and overall survival (OS) of patients with indicated cancers. (**B**) The association between RET mRNA expression and recurrence -free survival (RFS) of patients with indicated cancers. (**C**) The relation between RET amplification and patient OS in different cancers. (**D**) The association between RET somatic mutation and patient RFS in different cancers.

To investigate the clinical significance of RET expression in human cancers, we performed the correlation analysis between RET mRNA levels and patient OS and RFS in different cancer types. First, we found that high RET levels were positively associated with poor patient OS in nine cancer types including cervical squamous cell carcinoma and endocervical adenocarcinoma (CESC), esophageal squamous cell carcinoma (ESCA), HNSC, KIRC, KIRP, LICH, SARC, STAD and UCEC. On the contrary, high RET expression resulted in better OS in patients with esophageal carcinoma (ESCA), LUCA, pancreatic adenocarcinoma (PAAD), and THCA (Fig. 9A). Meanwhile, the correlation analysis between RET expression and patient RFS demonstrated that high RET expression correlated with shorter patient RFS in CESC, KIRP, SARC, STAD and UCEC (Fig. 9B). We further analyzed the relationship between patient OS and RET alterations. No significant differences were found between patient OS and overall RET alterations (Supplementary Fig. S4). However, it was suggested that higher rate of RET amplification might increase patient OS in HNSC (Fig. 9C), whereas mutations of RET were implicated in causing worse prognosis in SARC (Fig. 9D).

## Discussion

The RET aberrations through either gain-of-function mutations that result in constitutive receptor activation or through abnormal expression have been implicated in heritable and sporadic tumors^4,24–26^. Specifically, RET mutations activation leads to the upregulated downstream signaling, such as MAPK and PI3K pathways, and further promotes cell proliferation and survival^5,27–29^. To better understand the beneficial role of targeting RET in cancers, we have profiled the genomic landscape of RET alterations, mRNA expression and methylation across 32 TCGA human cancers in this study. Here, we reported there were approximately 3.0% of RET alterations among 10,967 TCGA cancers with somatic mutations in the other domains (172 cases) being the most frequent variant, followed by mutations in the PKinase_Tyr domain (63 cases), RET fusions (42 cases) and other alterations, respectively. Notably, SKCM, UCEC and THCA had the highest frequencies of RET aberrations.

The leading altered form in SKCM and UCEC was RET mutation occurring in the other and PKinase_Tyr domains, the latter of which affected the RET function to a greater extent. By using cBioPortal cancer genomics database, we further classified the somatic mutations into four categories: missense mutation, fusion, truncating mutation, and in-frame mutation. In SKCM, the most common mutation is G823E, which belongs to missense and locates in the PKinase_Tyr domain. There are so far no FDA-approved drugs for patients specifically with RET G823E mutant melanoma. In UCEC, 40 RET mutations were identified among total 529 cases. The most common one is RET S891L missense mutation. It’s at level 3b and considered likely oncogenic. Two FDA-approved RET inhibitors—pralsetinib and selpercatinib are therapeutically implied for the treatment of patients with RET S891L mutant UCEC^3,5^. Pralsetinib has been identified as a highly selective RET-kinase inhibitor by screening over 10,000 compounds^30^. It inhibited RET signaling and proliferation in both *in-vitro* RET-driven cancer cell lines and *in-vivo* RET-driven cancer models. Selpercatinib exerts potent suppressive activity against diverse RET-activating mutations. Importantly, in mouse tumor models, selpercatinib showed substantial control of diverse tumor growth^31,32^. Of note, their clinical utility in patients with RET S891L mutant UCEC still needs further investigation.

RET alterations have been found in diverse THCA subtypes—including papillary, follicular, medullary and anaplastic thyroid carcinomas^33^. Interestingly, mutations in RET protein have been previously considered as a hallmark of medullary thyroid carcinoma, since they were reported in 40–80% of medullary thyroid cancer patients and were highly associated with poor prognosis—in terms of more metastasis, larger tumor sizes and poorer overall survival^8,34–36^. By contrast, RET fusions mediated by gene rearrangements are more frequently observed in papillary thyroid cancer, which is the most common differentiated THCA subtype^37^. RET fusions can also be present in other THCA subtypes, such as poorly differentiated thyroid carcinomas and anaplastic thyroid carcinomas^38,39^. Indeed, in this study, THCA demonstrated the highest rate of RET fusions, which was mainly caused by the juxtapositions of 5' sequences from other genes with 3' sequences from RET^5,40^. The most frequent RET fusions in THCA are coiled-coil domain containing 6 (CCDC6)-RET with level 1 therapeutically, which mediates constitutive activation of the RET kinase and is thus known to be oncogenic^41–44^. The underlying mechanism for CCDC6–RET is a chromosome 10q paracentric inversion^45^. Both selpercatinib and pralsetinib have been FDA-approved for the treatment of patients with metastatic RET-fusion positive thyroid cancer^30^. Besides, there are also other FDA-approved inhibitors with anti-RET activity that are available for the treatment of RET-mutated thyroid tumors. For instance, in a double-blind phase III clinical trial, patients with advanced medullary thyroid carcinoma receiving cabozantinib have shown significant progression-free survival^11^.

Other cancer types, such as COADREAD, LUAD, LUSC, STAD, BRCA, HNSC and OV, also had relatively high RET alterations with predominant mutations in the other domains, whose function was not clear. LUAD and LUSC are the most common histological types of non-small cell lung cancer (NSCLC). Notably, RET rearrangements represent profound oncogenic drivers in approximately 1–2% of patients with NSCLC^46^. Here, we were also able to detect two RET fusions (CCDC6 and TRIM33) in patients with LUAD. CCDC6-RET is considered to regulate cell proliferation and cell growth mainly through RAS-ERK pathway^41,47^. CCDC6-RET fusion was associated with enhanced resistance to EGFR tyrosine kinase inhibitor in EGFR-mutant NSCLC cell lines via excessive cellular proliferation^43^. TRIM33, also called RFG7 or TIF1γ, consists of the transcription intermediary factor 1 family and regulates cellular differentiation^48^. It is caused by the replacement of 5’ portion of RET, which ultimately results in RET dimerization and the tyrosine kinase activation^9^. The RET-targeted inhibitors selpercatinib and pralsetinib are FDA-approved for the treatment of patients with metastatic RET-fusion positive NSCLC at level 1^49^. Besides, cabozantinib and vandetanib have been implied as new therapeutic targets and drug treatments in patients with RET fusion-positive lung cancers at level 2 and 3b, respectively^46,50,51^.

The RET M918T has been reported as a hotspot somatic mutant molecular in sporadic MTC (about 43%-71% of MTC cases) for decades, and is highly associated with poor prognosis of MTC patients^8,22,34,35,52^. It is caused by a single nucleotide change, which replaces methionine at position 918 with a threonine in the tyrosine kinase domain and further alters the ATP binding affinity and receptor activation regardless of RET dimerization^53–55^. In this study, we reported four RET M918T-altered cases in patients with PCPG, which are all considered to be oncogenic and have level-3B therapeutic implications. Although both selpercatinib and pralsetinib possess anti-RET M918T activity against THCA, their clinical therapeutic effects on patients with RET M918T mutant PCPG is unclear. It is also suggested that the M918T mutation could induce resistance against some drugs, such as cabozantinib, lenvatinib, vandetanib and motesanib^14^. It might be because its mutant site locates at distant C-terminal lobe away from the tyrosine kinase inhibitor binding site, which thus leads to a long-range effect on the construction of the inhibitor binding pocket. Also, activating RET M918T mutation is reported to acquire resistance against KRAS G12 C inhibition^12^. Hence, combination therapy strategies may be required to overcome the resistance of M918T mutation and improve its therapeutic efficiency. As co-occurring alterations commonly exist and affect the prognosis or therapeutic response of patients with RET aberrations to some extent^22^**,** we have also evaluated the alterations that co-occurred with RET in pan cancer. 273 significant co-aberrations have been identified among genes, most frequently in Notch signaling, TGF-β pathway, cell cycle, and Ras-Raf-MEK-Erk/JNK signaling. Additionally, the most commonly co-altered genes among 311 RET altered cases are TP53 (n = 162), PIK3CA (n = 84), PTEN (n = 79), and CDKN2A (n = 76), respectively. Though not all RET alterations are clearly oncogenic, multiple aberrations with putative driving roles in tumorigenesis, such as TP53 truncating sequence variant and PIK3CA missence sequence variant, co-exist in most missence RET-aberrant cases with unknow significance, unmarking potential actionable oncogenic effects of those RET missence sequence variants on cancers. Of note, RET-targeted therapies might be less efficacious in the setting of co-aberrations in its downstream pathways, like PI3K/AKT and RAS/MAPK.

Overexpression of RET has been found in diverse tumors including—but not limited to—melanoma, colorectal cancer, renal cell carcinoma, prostate cancer and head and neck tumors^23^. Increased RET level is seen in up to 65% of pancreatic ductal adenocarcinomas and is associated with advanced tumor progression and poor prognosis^5,56^. Using the GTEx portal database, we have identified BLCA, BRCA, HNSC, LUAD, PCPG, PRAD and THCA with increased RET expression compared to their corresponding normal tissues. Specifically, PCPG demonstrated the most upregulated RET expression cancer type. While RET CNV positively corelated with RET mRNA expression among 32 TCGA cancers, the mRNA expression of RET and its downstream pathway mediators was negatively associated with their methylation levels, indicating critical roles of CNV and methylation process in regulating RET transcription in cancers. The prognostic roles of RET expression were very clear in this dataset. Remarkably, higher RET levels resulted in poorer patient OS and RFS in CESC, KIRP, SARC, STAD and UCEC. The frequencies of RET alterations in UCEU (7.56%), STAD (4.32%) and SARC (3.53%) were relatively higher than CESC (1.01%) or KIRP (0.0%). In addition, mutation of RET was found to be positively related to worse prognosis in SARC, whereas higher rate of RET amplification could increase patient OS in HNSC.

Nevertheless, there were several limitations in the study. First, although RET alterations were profiled across a cohort of 32 human cancer samples, analysis based on these published data could be biased by the limited availability of some special and rare cancer types. Further studies recruiting more samples are needed. Second, some cancer types did not have sufficient sample size, leading to insufficient specimens submitted to NGS. Finally, the alteration frequencies of RET among diverse cancers varied from 0 to 10%. The low alteration frequency (< 1.05%) indeed made our work more challenging. However, despite all these limitations, the study provides us better comprehensive profile of RET aberrations in diverse cancers using cBioportal, compared to analysis in individual cancers. We next aimed to focus on investigating the mechanisms of acquired drug resistance to RET inhibitors and proposing better therapeutic strategies in the combination of anti-RET therapy with other blockades.

## Conclusions

In conclusion, we profiled a comprehensive pan cancer analysis of RET expression, methylation, aberration and clinical patient outcomes among 32 TCGA cancers. Most RET alterations are crucial for oncogenesis, while some others are indicated as potential therapeutic targets—particularly for thyroid cancer. At last, both RET mutations and amplification were significantly correlated to patient prognosis in certain cancers, providing clinical significance in the development of RET-targeted therapy.

## Materials and methods

### Comprehensive bioinformatic analysis using different tools

Mutations, CNVs, RET mRNA expression and the clinical datasets were downloaded from cBioportal, which is a portal that allows users to study genetic alterations across samples and genes, and link these to clinical outcomes^57^. cBioportal provides the comprehensive annotation of variants from different databases, including COSMIC, Cancer Hotspots method, OncoKB, CIViC, and My Cancer Genome. As for the CNV data, the log ratio value means: − 2 = deep deletion; − 1 = shallow deletion; 0 = diploid; 1 = gain; 2 = amplification. RET mRNA expression data in different cancers, defined as NormalizeExpressionLevels _allsampleref.py, was generated from normalized values with the reference population of all samples independent of sample diploid status. A total of 10,953 patients with 10,967 samples across 32 cancer types were analyzed (Supplementary Table S1). The mRNA expression data were log10 transformed. Besides cBioportal, RET fusion gene data were also downloaded from TCGA Fusion Gene Database, covering fusion genes predicted by PRADA analysis of RNA sequencing data among 32 TCGA cancer types^58^.

To analyze the co-occurring aberrations associated with RET alterations in diverse pathways, we selected cancer study “TCGA Pan Cancer Atlas Studies” in the web interface of cBioPortal and chose the gene list from indicated general oncogenic pathways together with RET. Significant co-occurrence in each oncogenic pathway was calculated by the statistical method Mutual Exclusivity Modules (MEMo) and presented in the Mutual Exclusivity tab through cBioPortal. An OncoPrint was used to visualize and summarize the alterations of RET and its co-aberrant genes.

RET expression in normal tissues was acquired from The Genotype-Tissue Expression (GTEx). GTEx collects transcriptome data of different tissue types from healthy individuals^59^. Besides, the RET mRNA levels were compared between tumor tissues and paired normal tissues in different TCGA cancer types or specific cancer subtypes using the “Gene_DE” module of TIMER2 (tumor immune estimation resource, version 2) method. The ‘Gene_DE Module’ provides an efficient way for users to compare the gene mRNA levels in cancers versus matched normal tissues among all TCGA cancers. The log2 [TPM (Transcripts per million) + 1] transformed data of mRNA level were used for the box plots here. TIMER2 is a common platform for systematical bioinformatic analysis of immune cells infiltration across diverse cancers^60^. Specifically, GSCALite is an online server, which provides comprehensive analysis including gene-targeted drug sensitivity, gene methylation, genomic variations and corelated survival analysis^61^. In this study, GSCALite platform was applied for studying differential methylation of RET and its downstream genes between tumor tissues and the coresponding normal tissues, and was also used for analyzing the association between methylation and the mRNA expression of RET and its downstream genes.

At last, we analyzed the correlation between the RET expression level and patient overall survival (OS) and recurrence-free survival (RFS) using the Kaplan–Meier Plotter, which is a public platform for investigating the roles of diverse genes in patient survival. Notably, Kaplan–Meier Plotter, established by a PostgreSQL server, automatically integrates the transcriptome data and clinical data from different databases including—but not limited to—Gene Expression Omnibus (GEO), The European Genome-phenome Archive (EGA) and TCGA^62^. Furthermore, we performed the survival analysis for RET alteration or amplification status using the clinical data downloaded from cBioportal. The patient OS and RFS were evaluated here. The hazard ratio and 95% confidence intervals were shown as forest plots.

Publicly available human data was used in this study, all methods were performed in accordance with the Declaration of Helsinki.

### Statistical analysis

The SPSS 12.0 software (IBM Analytics, USA) was used for statistical analysis. Student’s *t* test, linear regression and Cox regression analysis were performed when appropriate. *P* < 0.05 was considered statistically significant.

## Supplementary Information

Supplementary Figures.

Supplementary Table 1.

Supplementary Table 2.

Supplementary Table 3.

Supplementary Table 4.

## Data Availability

The datasets used and/or analysed during the current study available from the corresponding author on reasonable request.

## Abbreviations

RET: Rearranged during transfection
CNVs: Copy number variants
TCGA: The Cancer Genome Atlas
CCDC6: Coiled-coil domain containing 6
HD: Hirschsprung disease
GTEx: Genotype-tissue expression
TPM: Transcripts per million
OS: Overall survival
RFS: And recurrence-free survival
GEO: Gene expression omnibus
EGA: The European Genome-phenome archive
aa: Amino acid
