# Supplementary material for: An integrative pan cancer analysis of RET aberrations and their potential clinical implications
Source: Sci Rep. 2022 Aug 17;12:13913. doi: 10.1038/s41598-022-17791-y (PMC9386015; doi:10.1038/s41598-022-17791-y)
Supplement: Supplementary file 1 — Supplementary Figures. [file 41598_2022_17791_MOESM1_ESM.docx]

**
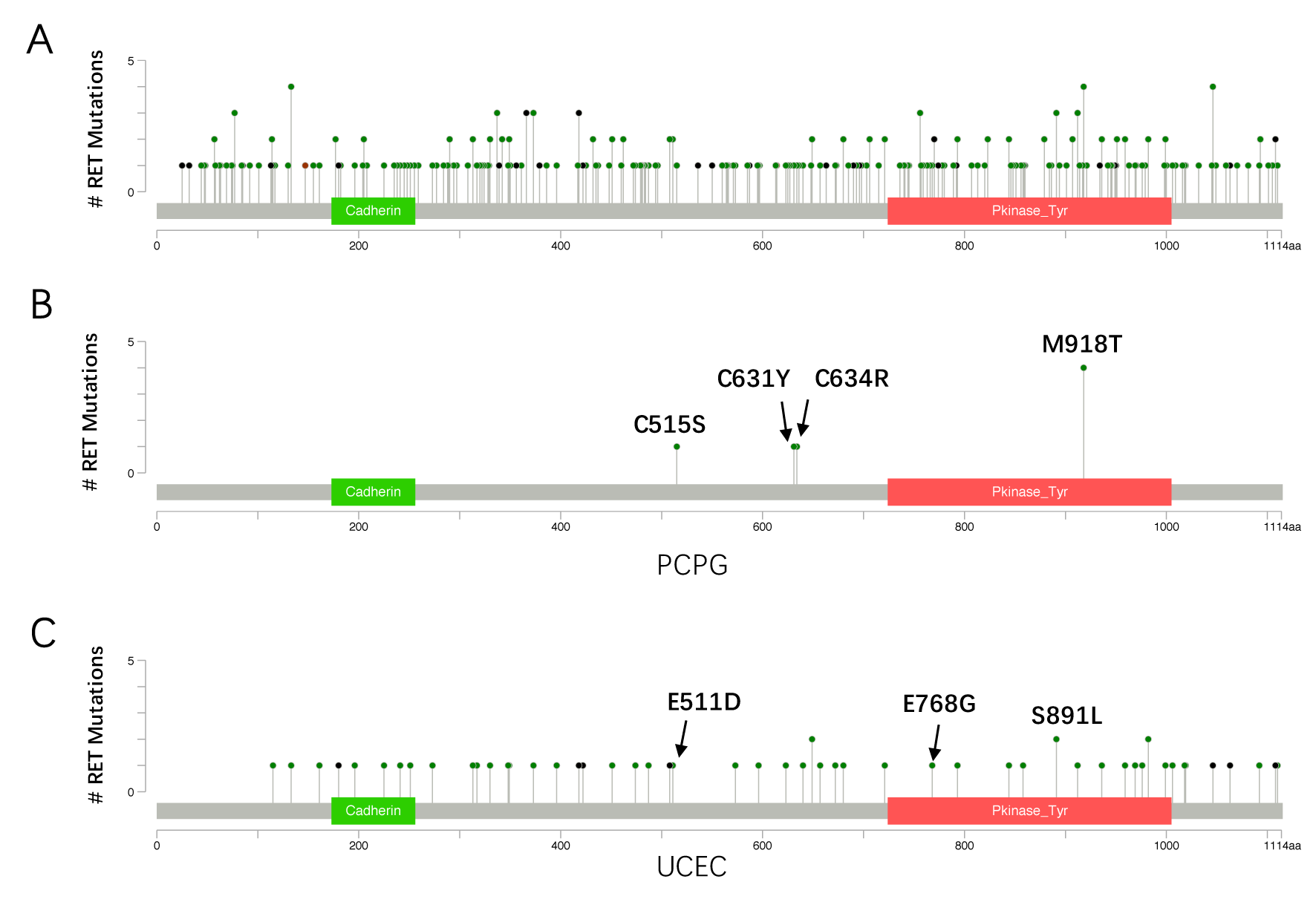
**

**Supplementary Figure S1.** The distribution of RET mutation in diverse functional domains. (A) The distribution of RET mutation in indicated functional domains in pan cancer. (B) The distribution of RET mutation in indicated functional domains in PCPG. (C) The distribution of RET mutation in indicated functional domains in UCEC.

**
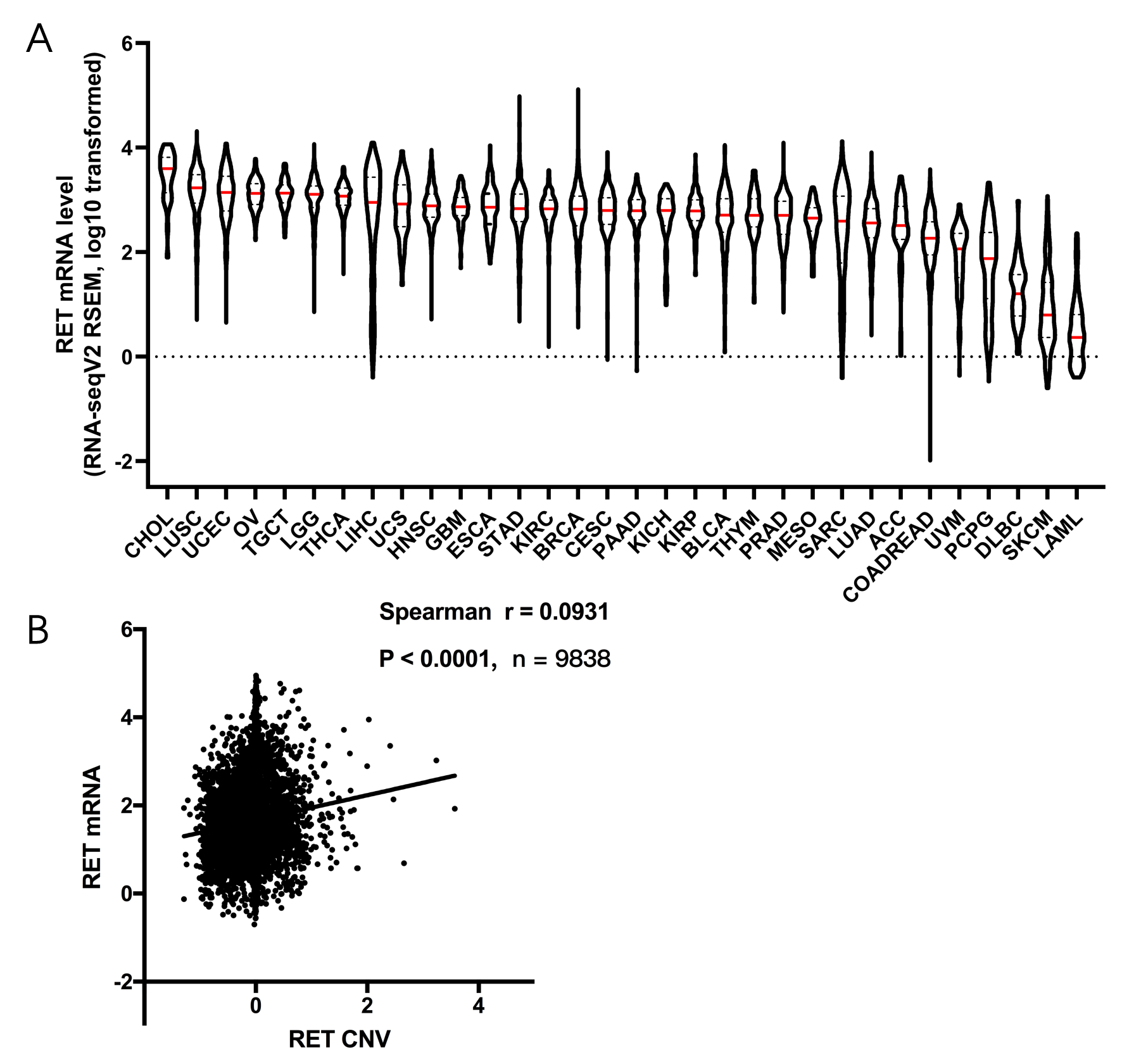
**

**Supplementary Figure S2.** RET mRNA levels and the correlation with RET CNVs among cancers. (A) RET mRNA levels (RNA-seqV2 RSEM, log10 transformed) in 32 types of TCGA cancers. (B) The association between RET linear copy number value and RET mRNA levels (RNA-seqV2 RSEM, log10 transformed) in 32 types of TCGA cancers.

**
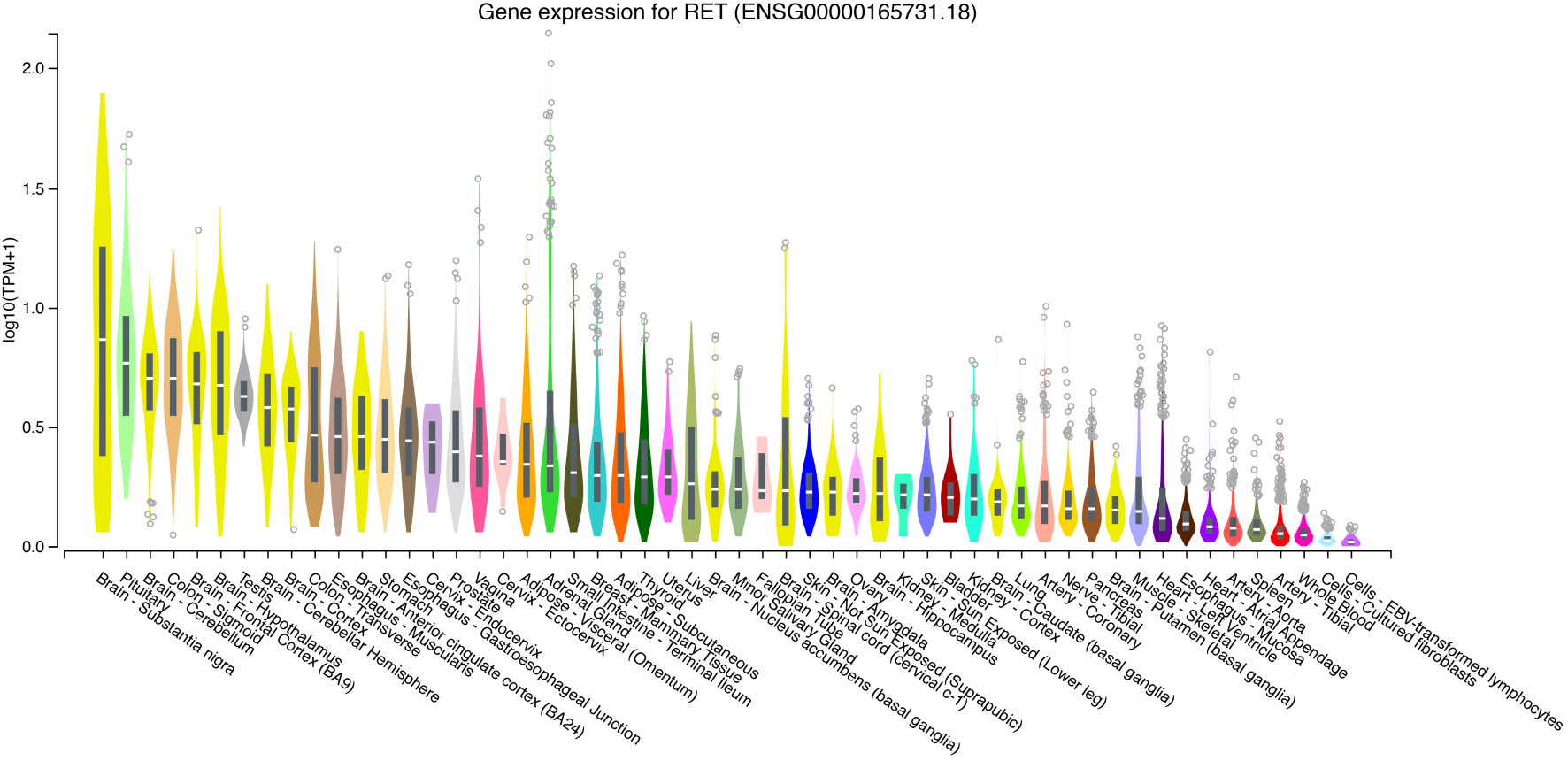
**

**Supplementary Figure S3.** RET mRNA levels (log10, TPM +1 transformed) in non-tumor tissues from GTEx portal database.

**
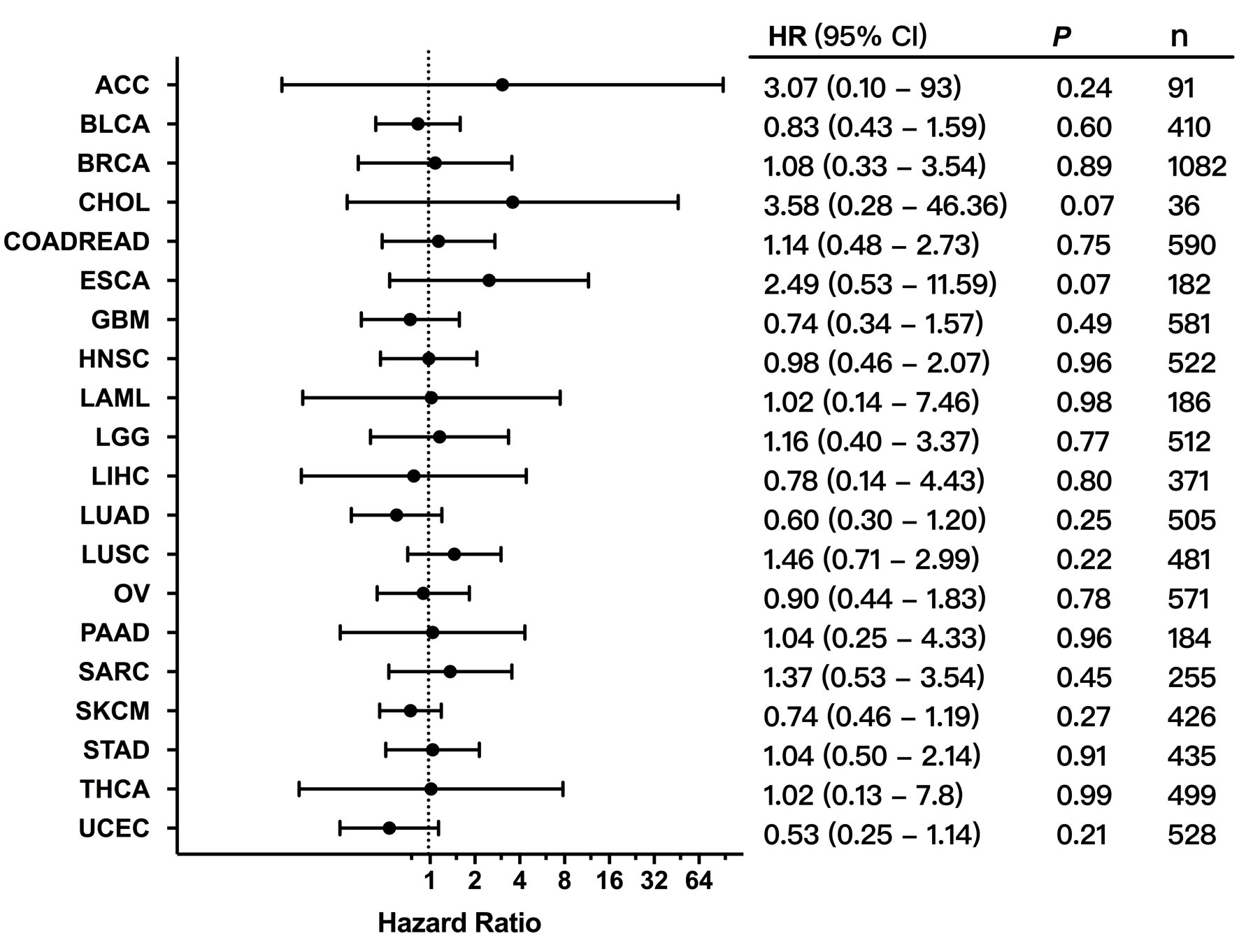
**

**Supplementary Figure S4.** The correlation between RET alterations (mutations and CNVs) and patient OS in different cancers.

**Table Legends**

**Supplementary Table S1.** Summary of TCGA cancer types and case numbers.

**Supplementary Table S2.** Detailed information of 311 human RET somatic mutations in 32 cancer types from cBioportal.

**Supplementary Table S3.** Somatic mutation distribution in different RET functional domains for RET-mutant cancers and pan cancer.

**Supplementary Table S4.** Lists of co-occurring aberrations associated with RET alterations in oncogenic pathways in pan cancer.
